# Body image among bullied obese children: an Egyptian case-control study

**DOI:** 10.1186/s12889-026-26346-z

**Published:** 2026-02-21

**Authors:** Nermine N. Mahfouz, Mona A. Elabd, Azza Abd El-Shaheed

**Affiliations:** Department of Child Health, Medical Research and Clinical Studies Institute, Medical Research Centre of Excellence, National Research Centre, Cairo, Affilition, 60014618 ID Egypt

**Keywords:** Childhood obesity, Bullying experiences, Family refusal, Body image dissatisfaction

## Abstract

**Background:**

Childhood obesity is a rising worldwide health issue with significant physical, psychological, and social implications. Beyond physical health, obesity has profound psychological effects, particularly on body image perception and self-esteem, which can be exacerbated by bullying and social stigmatization.

**Aim:**

To assess body image perception among bullied obese children in Egypt and to examine the relationship between obesity, bullying experiences, and body image dissatisfaction.

**Methods:**

This case-control study was conducted on 86 Egyptian children aged 5 to 10 years who attended the outpatient nutrition and immunity clinic at the Medical Research Centre of Excellence (MRCE). A comprehensive, structured questionnaire and clinical examination were used and analyzed to assess participants.

**Results:**

The study included 86 children; 40 males and 46 females. Out of 86 children, 44 children were obese with mean of age (8.16 ± 1.78 y) and 42 were healthy children as controls with mean of age (7.69 ± 1.8. 55 y). A total of 27 children (34.6%) in the study population displayed acanthosis nigricans. Students in the obesity group reported higher rates of school bullying (36.4%) as compared to controls (11.9%). The family bullying was significantly higher in the obesity group at 40.9% compared to the control group at 7.1% (*p* = 0.001). A significant difference (*p* = 0.000) in sadness feeling was reported in obese children (56.8%) as compared with controls (11.9%). Children with family refusal showed statistically significant higher occurrences of expressing low self-esteem by refusing to be photographed (50%) and showing sadness (75%), especially in females.

**Conclusion:**

This study highlights the obesity and its association with obesity-related health risks and emotional distress. School refusal was strongly linked to bullying and academic pressure. Negative school interactions affected family relationships, while family denial increased bullying and emotional distress. Females showed greater vulnerability to sadness than males.

**Supplementary Information:**

The online version contains supplementary material available at 10.1186/s12889-026-26346-z.

## Introduction

Childhood obesity is a rising worldwide health issue with significant physical, psychological, and social implications. The World Health Organization (WHO) reports a substantial increase in childhood obesity over the last forty years, with around 39 million children under five years old categorized as overweight or obese in 2020 [1]. Obese children have a heightened risk of developing serious health problems, including type 2 diabetes, cardiovascular disease, and metabolic disorders [2]. Beyond its physical health burden, obesity exerts profound psychological effects, particularly on body image perception and self-esteem, which are often intensified by bullying and social stigmatization [3].

Weight-based teasing is one of the most common forms of peer victimization in school settings, and obese children are disproportionately targeted compared to their non-obese peers [4, 5]. Such experiences can lead to negative body image, increased risk of depression and anxiety, and the development of disordered eating behaviors [6]. Repeated exposure to weight-related bullying may cause children to internalize negative societal attitudes toward body weight, resulting in low self-worth and emotional distress [7]. This relationship is particularly concerning in low- and middle-income countries where public awareness, psychological support, and mental health resources are limited [8].

In recent years, Egypt has mirrored global trends in the increasing prevalence of childhood obesity [9]. National data indicate alarming rates of overweight and obesity among Egyptian children—19.1% of girls and 15.7% of boys aged 5–19 years in 2016, with projections reaching 21% and 17.2%, respectively, by 2019 [10]. Environmental and cultural factors such as unhealthy dietary habits, sedentary lifestyles, and inadequate physical activity contribute to this growing problem. Moreover, obesity-related stigma may foster bullying and body image concerns among Egyptian children, influenced by social and cultural ideals surrounding appearance and weight [11]. Despite global evidence that obesity-related bullying has severe consequences for mental health and quality of life [12], research exploring these psychosocial impacts within the Egyptian context remains scarce.

This study draws upon social stigma theory, which posits that socially devalued attributes, such as excess body weight, may lead to internalized shame, reduced self-esteem, and maladaptive social interactions [13]. Applying this perspective allows for a deeper understanding of the psychological consequences of obesity in childhood and its interaction with bullying experiences.

This study aimed to assess body image perception among bullied obese children in Egypt and to examine the relationship between bullying experiences, body image dissatisfaction, and psychological outcomes.

## Methodology

### Study design

This case–control study was conducted on 86 Egyptian children aged 5 to 10 years who attended the outpatient nutrition and immunity clinic at the Medical Research Centre of Excellence (MRCE), Dokki, Giza Governorate. The MRCE is a tertiary-level public research and clinical facility that provides specialized pediatric and metabolic services for the Cairo–Giza metropolitan region, enabling access to a wide sociodemographic range of patients.

Data were collected between March 2023 and February 2024, ensuring adequate seasonal representation.

Ethical approval for the study was obtained from the Ethical Committee of the National Research Centre (NRC) in accordance with the Declaration of Helsinki. Written informed consent was secured from the guardians of the participating children before enrollment.

### Study population and grouping

The study population was categorized into two groups:

- *Group 1 (Obese)*: 44 obese children diagnosed with simple obesity (Body Mass Index (BMI) ≥ 95th percentile for age and sex), assessed and plotted using *CDC growth curves*.
- CDC references were adopted as the most widely validated international tool applicable in comparative epidemiological studies.
- *Group 2 (Control)*: 42 normal-weight healthy children, with BMI ranging from the 5th to 84th percentile according to CDC growth standards [14].

A stratified random sampling technique was used to ensure balanced representation of children across age and gender categories. The strata were defined according to two age groups (5–7 years and 8–10 years) and sex (male and female). Within each stratum, children attending the outpatient nutrition and immunity clinic at the Medical Research Centre of Excellence were screened for eligibility and categorized according to their body mass index (BMI) into obese or normal-weight groups based on CDC growth standards.

Within each stratum, participants were then randomly selected using a simple random sampling method, applying computer-generated random numbers (Microsoft Excel RAND function) to identify eligible children from the clinic records. This approach ensured equal opportunity of selection and representativeness of both obese and non-obese children across age and gender strata, minimizing potential selection bias.

### Sample size calculation and selection

The sample size was calculated to detect a statistically significant difference in the proportion of children reporting sadness—a key psychosocial outcome—between obese and non-obese groups. Based on previous findings, it was estimated that 56.8% of obese children and 11.9% of non-obese controls would report frequent sadness. The calculation followed Fleiss [15].

and was performed using G*Power 3.1.9.7, assuming a two-sided 95% confidence level, 80% power, and an effect size (w) = 0.45, derived from these proportions, and the effect size (Cohen’s h) was 1.01; representing a very large difference between groups.

The sample size calculation is summarized as follows:

**Table:** 

Proportion (Obese Group)	Proportion (Control Group)	Confidence Level	Power	Allocation Ratio	Effect size (h)	Required Sample Size (per group)	Total Sample Size Required
0.568	0.119	95%	80%	1:1	1.01	28	56

No pilot data or previous Egyptian studies were available to refine these assumptions.

To increase statistical power and allow subgroup analyses, the total sample was expanded to 86 children (44 cases, 42 controls).

### Inclusion and exclusion criteria

#### Inclusion criteria

Egyptian children of both sexes aged 5–10 years, meeting group-specific BMI criteria.

#### Exclusion criteria

children below 5 or above 10 years, non-Egyptian children, and those with endocrine-related (e.g., Cushing syndrome) or syndromic obesity (e.g., Prader–Willi syndrome). Secondary causes of obesity were excluded through medical history review, clinical examination, and basic diagnostic tests when clinically indicated. Health status for controls was verified by reviewing recent medical records and confirming normal findings during current clinical examination.

### Research tools and data collection

A comprehensive, structured questionnaire was designed specifically for this study and uploaded in English as supplementary material. The instrument was adapted from the *Edmonton Obesity Staging System for Pediatrics* [16] and underwent bilingual translation (English ↔ Arabic), cultural adaptation, and pilot testing for content validity. Expert pediatricians and psychologists reviewed the translated version to ensure conceptual accuracy and cultural relevance.

Pilot testing confirmed clarity and internal consistency before field use.

Likert-type scales (five-point) were used for attitudinal and psychological questions, whereas dichotomous yes/no responses were applied to factual or behavioral items, allowing both nuanced and categorical data capture.

Clinical examinations were performed by *trained personnel*,* and intra- and inter-rater reliability was periodically checked* to minimize measurement error.

The questionnaire included:

- *Demographic and Family History*: parental consanguinity, smoking, family history of obesity, hypertension, and diabetes mellitus.
- *Dietary and Lifestyle Assessment*: food frequency, eating behaviors, and physical-activity levels.
- *Psychological and Mental Health Assessment*: body-image perception, school refusal, ADHD symptoms, family support, and exposure to bullying or teasing.
- *Clinical Examination*: skin inspection for acanthosis nigricans, blood-pressure measurement using a calibrated sphygmomanometer (Accoson, London, UK), and anthropometric indices (weight, height, BMI, waist–hip and waist–height ratios).

#### Statistical analysis

- All data were coded, entered, and analyzed using IBM SPSS Statistics version 28.0 (IBM Corp., Armonk, NY, USA). The significance level was set at *p* < 0.05.

#### Missing data

- Data completeness was verified prior to analysis. Minor missing values were observed in certain variables (e.g., blood pressure and acanthosis nigricans) due to unrecorded measurements during clinical examination. These cases were excluded listwise and not imputed; a complete-case analysis approach was applied, with comparisons performed on available data only.
- Because the study compared two clearly defined groups (obese and non-obese children) that were matched by age and sex and stratified during sampling, no additional statistical adjustment for confounding was deemed necessary. Comparisons between participants with and without missing data showed no significant differences in age, sex, or BMI, suggesting that the missingness was random and unlikely to introduce bias.

1. Descriptive Statistics:
  Quantitative variables were tested for normality using the Shapiro–Wilk test and visual inspection of histograms and Q–Q plots.
  Normally distributed data were expressed as mean ± standard deviation (SD), while non-normally distributed data were presented as median and interquartile range (IQR).
  Categorical variables were summarized as frequencies and percentages.
2. Inferential Tests
  Comparisons between two independent groups (obese and non-obese children) were performed using the independent-samples t-test for normally distributed variables and the Mann–Whitney U test for nonparametric data.
  Associations between categorical variables were assessed using the Chi-square test or Fisher’s exact test when Chi-square assumptions were not met (i.e., expected counts <5 in more than 20% of cells). Effect sizes were expressed as odds ratios (ORs) with 95% confidence intervals.
  Correlations between quantitative variables were examined using Pearson’s correlation coefficient for parametric data and Spearman’s rho for nonparametric data.
  Missing data were handled by pairwise deletion after verifying that omissions were random and < 5 % of the dataset.
  Potential confounders such as age and sex were examined through stratified analyses.
3. Diagnostic Accuracy
  Receiver Operating leftacteristic (ROC) curve analysis was performed to assess the diagnostic performance of selected biochemical parameters including serum vitamin D and serum zinc, in differentiating obese children with negative psychosocial outcomes (such as sadness or low self-esteem) from those without such outcomes.
  The Area Under the Curve (AUC) was calculated to evaluate the overall discriminative ability of each marker. The optimal cut-off value for each biomarker was determined using Youden’s index, which maximizes the sum of sensitivity and specificity. The corresponding sensitivity, specificity, positive predictive value (PPV), and negative predictive value (NPV) were also reported with 95% confidence intervals.
  Because multiple comparisons were performed, the potential for *Type I error inflation* was acknowledged. Given the study’s exploratory design and modest sample size, no formal adjustment (e.g., Bonferroni or Benjamini–Hochberg correction) was applied. Therefore, *p*-values should be interpreted with caution, considering the overall pattern and strength of associations rather than individual significance thresholds.

A p-value < 0.05 was considered statistically significant.

Diagnostic leftacteristics was computed as follows:

- Sensitivity = $$\left(\mathrm{True}\;\mathrm{positive}\;\mathrm{test}/\mathrm{Total}\;\mathrm{positive}\;\mathrm{golden}\right)\times100$$ 
- Specificity = $$\left(\mathrm{Total}\;\mathrm{negative}\;\mathrm{test}/\mathrm{Total}\;\mathrm{negative}\;\mathrm{golden}\right)\times100$$ 
- Diagnostic accuracy = 
  $$\frac{\left(\mathrm{True}\;\mathrm{positive}\;\mathrm{test}+\mathrm{True}\;\mathrm{negative}\;\mathrm{test}\right)\times100}{\mathrm{Total}\;\mathrm{cases}}$$
- $$\mathrm{Youden}'\mathrm s\;\mathrm{index}=\mathrm{sensitivity}+\mathrm{specify}-1$$ 
- Positive predictive value (PPV) =
  $$\frac{\mathrm{True}\;\mathrm{positive}\;\mathrm{test}\times100}{\mathrm{Total}\;\mathrm{positive}\;\mathrm{test}}$$
- Negative predictive value (NPV) =
  $$\frac{\mathrm{True}\;\mathrm{negative}\;\mathrm{test}\times100}{\mathrm{Total}\;\mathrm{negative}\;\mathrm{test}}$$

The internal consistency of the psychological and psychosocial domains of the questionnaire was assessed using Cronbach’s alpha on the study sample. The scale demonstrated acceptable reliability (Cronbach’s α = 0.71).

## Results

The research population included 40 male participants, whose proportion is 46.5%, while 46 females comprise the remaining 53.5% of the cohort. The study design contained 44 obese children and 42 non-obese children as controls for balanced comparison purposes. Results indicated that the mean ± SD BMI was 26.33 ± 4.63 kg/m² in obese children and 16.65 ± 2.57 kg/m² in controls. The corresponding waist-to-height ratio (WHTR) values were 0.60 ± 0.06 and 0.47 ± 0.05, respectively (*p* = 0.000 for both). The waist-to-hip ratio (WHR) showed a median (Q1–Q3) of 0.90 (0.87–0.93) among obese participants and 0.87 (0.83–0.90) among controls (*p* = 0.000). Mean ± SD BMI was 21.83 ± 5.41 kg/m² in boys and 21.41 ± 6.77 kg/m² in girls (*p* = 0.752).

The mean waist-to-height ratio (WHtR) was 0.55 ± 0.08 for boys and 0.53 ± 0.08 for girls (*p* = 0.183).

Median waist-to-hip ratio (WHR) was 0.90 [IQR 0.87–0.93] in boys and 0.87 [IQR 0.83–0.90] in girls, showing no significant difference (*p* > 0.05).

About 55 children (64.0%) possess a WHTR value equal to or higher than 0.5, which signifies increased central obesity risk, but 31 children (36.0%) exhibit WHTR below 0.5. 27 children (31.4%) displayed abdominal obesity as shown by their WHR at or above the 95th percentile, but 59 children (68.6%) have values below this cutoff. In the study population, 27 children (34.6%) displayed acanthosis nigricans, although 51 children (65.4%) did not present with this skin issue.

Blood pressure measurements were available for 71 of 86 participants (82.6%), with 15 missing due to unrecorded values. Acanthosis nigricans was observed in 34.6% (27/78) of participants with data available, as 8 cases had missing records.

The demographic and clinical leftacteristics presented in Table (1) show a relatively balanced distribution between males (46.5%) and females (53.5%), as well as between cases (51.2%) and controls (48.8%), minimizing potential gender or group bias. Regarding anthropometric indices, a notable proportion of participants had elevated central adiposity, with 64.0% exhibiting a waist-to-height ratio (WHTR) ≥ 0.5, while 31.4% exceeded the 95th percentile in waist-to-hip ratio (WHR). These findings suggest a substantial prevalence of obesity-related risk factors in the study population. Blood pressure distribution indicates that the majority (93.0%) had normal values, while only 7% fell into elevated or hypertensive categories, though the small numbers in these subgroups may limit meaningful interpretation. The presence of acanthosis nigricans, a marker of insulin resistance, was reported in 34.6% of participants, aligning with the observed high prevalence of central obesity (Table 1 and 2).

**Table 1:** Frequency distribution of the study group leftacteristics (Expressed in number and Percentage)

Variables	Categories	Frequency	Percent (%)
Gender	Male	40	46.5
	Female	46	53.5
Case/Control	Case	44	51.2
	Control	42	48.8
Waist/Height Ratio (WHTR)	< 0.5	31	36.0
	≥ 0.5	55	64.0
Waist/Hip Ratio (WHR)	< 95th percentile	59	68.6
	≥ 95th percentile	27	31.4
Blood Pressure Class	Normal Blood Pressure	66	93.0
	Elevated Blood Pressure	2	2.8
	Stage 1 Hypertension	2	2.8
	Stage 2 Hypertension	1	1.4
	Total	71	100.0
	Missing	15	
Acanthosis Nigricans	Yes	27	34.6
	No	51	65.4
	Total	78	100.0
	Missing	8	

The comparison between cases and controls regarding school and family-related complaints reveals that most variables did not differ significantly between groups, suggesting broadly similar experiences in educational and family settings. School-related complaints, including reading and math difficulties, refusal of school, and bullying at school, showed no statistically significant differences, although bullying in school approached significance (*p* = 0.068), with a higher proportion of cases (36.4%) reporting bullying compared to controls (11.9%).

The most notable finding was the significantly higher prevalence of family bullying among cases (40.9%) compared to controls (7.1%), with a strong statistical association (*p* = 0.001). This highlights family bullying as a potentially critical differentiating factor between groups. Other complaints, such as refusal of family or refusal of play, showed no significant associations.

**Table 2:** Comparison between cases and controls as regards school and family related complaints:

		case/control		Total (*n* = 86) n (%)	χ² (df)	*p* value	OR (95% CI)	χ² (df)	*p* value
		Case (*n* = 44) n (%)	Control (*n* = 42) n (%)						
School complains	Yes	6 (13.6%)	7 (16.7%)	13 (15.1%)	1.266	0.867	‎0.79 ‎‎(0.24 – ‎‎2.58)‎	0.15 (1)	0.695
	No	36 (81.8%)	32 (76.2%)	68 (79.1%)					
	Sometimes	1 (2.3%)	1 (2.4%)	2 (2.3%)					
	I do not know	1 (2.3%)	1 (2.4%)	2 (2.3%)					
	I do not go to school	0 (0%)	1 (2.4%)	1 (1.2%)					
Reading complains	Yes	7 (15.9%)	8 (19%)	15 (17.4%)	3.466	0.483	1.24 (0.41–3.80)	0.15 (1)	0.701
	No	35 (79.5%)	28 (66.7%)	63 (73.3%)					
	Sometimes	2 (4.5%)	4 (9.5%)	6 (7.0%)					
	I do not know	0 (0%)	1 (2.4%)	1 (1.2%)					
	I do not go to school	0 (0%)	1 (2.4%)	1 (1.2%)					
Math complains	Yes	5 (11.4%)	5 (11.9%)	10 (11.6%)	3.470	0.483	1.05 (0.28–3.94)	0.01 (1)	0.938
	No	38 (86.4%)	32 (76.2%)	70 (81.4%)					
	Sometimes	1 (2.3%)	3 (7.1%)	4 (4.7%)					
	I do not know	0 (0%)	1 (2.4%)	1 (1.2%)					
	I do not go to school	0 (0%)	1 (2.4%)	1 (1.2%)					
Refusal of school	Yes	9 (20.5%)	2 (4.8%)	11 (12.8%)	5.926	0.205	0.19 (0.04–0.96)	4.74 (1)	0.050
	No	32 (72.7%)	38 (90.5%)	70 (81.4%)					
	Sometimes	1 (2.3%)	1 (2.4%)	2 (2.3%)					
	I do not know	1 (2.3%)	0 (0%)	1 (1.2%)					
	I do not go to school	1 (2.3%)	1 (2.4%)	2 (2.3%)					
Bullying in school	Yes	16 (36.4%)	5 (11.9%)	21 (24.4%)	7.119	0.068	4.24 (1.38–13.0)	6.965 (1)	0.012*
	No	26 (59.1%)	34 (81%)	60 (69.8%)					
	Sometimes	1 (2.3%)	2 (4.8%)	3 (3.5%)					
	I do not know	0 (0%)	0 (0%)	0 (0%)					
	I do not go to school	1 (2.3%)	1 (2.4%)	2 (2.3%)					
Refusal of family	Yes	6 (13.6%)	2 (4.8%)	8 (9.3%)	3.171	0.205	0.32 (0.06–1.67)	2.01 (1)	0.266
	No	35 (79.5%)	39 (92.9%)	74 (86%)					
	Sometimes	3 (6.8%)	1 (2.4%)	4 (4.7%)					
	I do not know	0 (0%)	0 (0%)	0 (0%)					
	I do not go to school	0 (0%)	0 (0%)	0 (0%)					
Bullying in family	Yes	18 (40.9%)	3 (7.1%)	21 (24.4%)	15.156	0.001*	9.0 (2.4–33.3)	13,275 (1)	0.000*
	No	22 (50%)	37 (88.1%)	59 (68.6%)					
	Sometimes	4 (9.1%)	2 (4.8%)	6 (7%)					
	I do not know	0 (0%)	0 (0%)	0 (0%)					
	I do not go to school	0 (0%)	0 (0%)	0 (0%)					
Refusal of play	Yes	2 (4.5%)	1 (2.4%)	3 (3.5%)	1.288	0.732	0.51 (0.05–5.87)	0.30 (1)	0.584
	No	40 (90.9%)	40 (95.2%)	80 (93%)					
	Sometimes	1 (2.3%)	1 (2.4%)	2 (2.3%)					
	I do not know	1 (2.3%)	0 (0%)	1 (1.2%)					
	I do not go to school	0 (0%)	0 (0%)	0 (0%)					
Bullying pears	***Yes***	4 (9.1%)	2 (4.8%)	6 (7%)	1.955	0.582	0.50 (0.09–2.89)	0.62 (1)	0.677
	No	38 (86.4%)	38 (90.5%)	76 (88.4%)					
	Sometimes	2 (4.5%)	1 (2.4%)	3 (3.5%)					
	I do not know	0 (0%)	1 (2.4%)	1 (1.2%)					
	I do not go to school	0 (0%)	0 (0%)	0 (0%)					

*p < 0.05; χ² (df) = Pearson Chi-square test statistic with degrees of freedom, tests are Chi-square or Fisher’s exact where appropriate; percentages calculated from available data. *OR* odds ratio, *CI* confidence interval

The findings presented in Table (3) highlight both significant and non-significant differences between cases and controls regarding self-evaluated (SE) photo perception, sadness, and harmony. While a higher proportion of cases (20.5%) than controls (7.1%) reported positive SE photo perception, this difference did not reach statistical significance (*p* = 0.130), indicating that self-image based on photographs may not be a distinguishing factor between groups. Conversely, sadness demonstrated a highly significant difference (*p* < 0.001), with more than half of the cases (56.8%) reporting sadness compared to only 11.9% among controls. This finding suggests that sadness may be a meaningful emotional marker differentiating cases from controls. In contrast, harmony with parents/colleagues showed nearly identical distributions between cases (81.8%) and controls (83.3%), with no significant difference (*p* = 0.961), suggesting this factor is stable across groups. Overall, the results indicate that sadness is a salient emotional distinction, while self-perception and harmony do not differ substantially.

Bullying experiences at school were reported by 16 of 44 obese children (36.4%) compared with 5 of 42 controls (11.9%), showing a statistically significant association (*χ²* = 6.965, *p* = 0.012).

The odds of school bullying were approximately 4.2 times higher among obese children compared with controls (*OR = 4.24; 95% CI: 1.38–13.0*).

Bullying by family members was reported in 18 of 44 obese children (40.9%) and 3 of 42 controls (7.1%), showing a statistically significant association *(χ² = 13.275*,*p* = 0.000). The odds of experiencing family bullying were approximately 9 times higher among obese children compared with controls (*OR = 9.0; 95% CI: 2.4–33.3*).

**Table 3:** Comparison between cases and controls of the study group as regards SE photo, sadness, and harmony (expressed in number and percentage):

		case/control		Total (*n* = 86)	χ² (df)	*p* value	OR (95% CI)	χ² (df)	*p* value
		Case (*n* = 44)	Control (*n* = 42)						
SE photo	Yes	9 (20.5%)	3 (7.1%)	12 (14%)	4.097	0.130	0.30 (0.08–1.19)	3.17 (1)	0.188
	No	35 (79.5%)	38 (90.5%)	73 (84.9%)					
	Sometimes	0 (0%)	1 (2.4%)	1 (1.2%)					
	I do not know	0 (0%)	0 (0%)	0 (0%)					
	I do not go to school	0 (0%)	0 (0%)	0 (0%)					
SE sad	Yes	25 (56.8%)	5 (11.9%)	30 (34.9%)	19.115	0.000*	9.7; (3.2–29.4)	19,083 (1)	0.000*
	No	15 (34.1%)	30 (71.4%)	45 (52.3%)					
	Sometimes	4 (9.1%)	7 (16.7%)	11 (12.8%)					
	I do not know	0 (0%)	0 (0%)	0 (0%)					
	I do not go to school	0 (0%)	0 (0%)	0 (0%)					
Harmony P/C	Yes	36 (81.8%)	35 (83.3%)	71 (82.6%)	0.079	0.961	1.11 (0.36–3.39)	0.03 (1)	0.853
	No	3 (6.8%)	3 (7.1%)	6 (7%)					
	Sometimes	5 (11.4%)	4 (9.5%)	9 (10.5%)					
	I do not know	0 (0%)	0 (0%)	0 (0%)					
	I do not go to school	0 (0%)	0 (0%)	0 (0%)					

*p < 0.05; χ² (df) = Pearson Chi-square test statistic with degrees of freedom, tests are Chi-square or Fisher’s exact where appropriate; percentages calculated from available data. *OR* odds ratio, *CI* confidence interval

Table (4) demonstrates important associations between school refusal behaviors and several psychosocial and health-related factors. Statistically significant relationships were observed for bullying by peers (*p* < 0.001), bullying in school (*p* < 0.001), SE photo (*p* = 0.001), and SE sadness (*p* = 0.013), indicating that these variables are strongly associated with refusal to attend school. For example, almost all children reporting bullying in school or experiencing sadness/self-exposure behaviors also showed higher levels of school refusal. In contrast, bullying within the family, harmony with parents/children, and acanthosis nigricans did not reach statistical significance, suggesting that while they may play a role, their influence was less evident in this sample.

The prevalence of *SE sadness* was significantly higher among obese children (25/44; 56.8%) compared with controls (5/42; 11.9%) (*χ²* = 19.083, *p* = 0.000). The odds of sadness were 9.7 times higher in obese children than in controls (OR = 9.7; 95% CI = 3.2–29.4).

**Table 4:** Comparison between factors affecting school refusal behaviors in regard bullying patterns, SE photo, sadness, harmony and the presence of acanthosis nigricans (expressed in number and percentage):

		Refusal of school					X^2^	*p* value	OR (95% CI)	χ² (df)	p value
		Yes (*n* = 11)	No (*n* = 70)	Sometimes (*n* = 2)	I do not know (*n* = 1)	I do not go to school (*n* = 2)					
Bullying in family	Yes	5 (45.5%)	13 (18.6%)	2 (100%)	1 (100%)	0 (0%)	14.699	0.065	10.14 (2.07–49.67)	10.95 (1)	0.001*
	No	6 (54.5%)	51 (72.9%)	0 (0%)	0 (0%)	2 (100%)					
	Sometimes	0 (0%)	6 (8.6%)	0 (0%)	0 (0%)	0 (0%)					
	I do not know	0 (0%)	0 (0%)	0 (0%)	0 (0%)	0 (0%)					
	I do not go to school	0 (0%)	0 (0%)	0 (0%)	0 (0%)	0 (0%)					
Bullying peers	Yes	3 (27.3%)	0 (0%)	1 (50%)	1 (100%)	1 (50%)	51.310	0.000*	9.00 (1.55–52.27)	8.01 (1)	0.005*
	No	8 (72.7%)	67 (95.7%)	0 (0%)	0 (0%)	1 (50%)					
	Sometimes	0 (0%)	2 (2.9%)	1 (50%)	0 (0%)	0 (0%)					
	I do not know	0 (0%)	1 (1.4%)	0 (0%)	0 (0%)	0 (0%)					
	I do not go to school	0 (0%)	0 (0%)	0 (0%)	0 (0%)	0 (0%)					
Bullying in school	Yes	10 (90.9%)	8 (11.4%)	2 (100%)	1 (100%)	0 (0%)	128.353	0.000*	58.18 (6.76–500.92)	30.21 (1)	0.000*
	No	1 (9.1%)	59 (84.3%)	0 (0%)	0 (0%)	0 (0%)					
	Sometimes	0 (0%)	3 (4.3%)	0 (0%)	0 (0%)	0 (0%)					
	I do not know	0 (0%)	0 (0%)	0 (0%)	0 (0%)	0 (0%)					
	I do not go to school	0 (0%)	0 (0%)	0 (0%)	0 (0%)	2 (100%)					
SE photo	Yes	5 (45.5%)	4 (5.7%)	2 (100%)	0 (0%)	1 (50%)	27.800	0.001*	8.10 (1.96–33.46)	10.43 (1)	0.001*
	No	5 (54.5%)	65 (92.9%)	0 (0%)	1 (100%)	1 (50%)					
	Sometimes	0 (0%)	1 (1.4%)	0 (0%)	0 (0%)	0 (0%)					
	I do not know	0 (0%)	0 (0%)	0 (0%)	0 (0%)	0 (0%)					
	I do not go to school	0 (0%)	0 (0%)	0 (0%)	0 (0%)	0 (0%)					
SE sad	Yes	8 (72.7%)	20 (28.6%)	1 (50%)	0 (0%)	1 (50%)	19.322	0.013*	6.42 (1.56–26.50)	7.95 (1)	0.005*
	No	2 (18.2%)	42 (60%)	0 (0%)	0 (0%)	1 (50%)					
	Sometimes	1 (9.1%)	8 (11.4%)	1 (50%)	1 (100%)	0 (0%)					
	I do not know	0 (0%)	0 (0%)	0 (0%)	0 (0%)	0 (0%)					
	I do not go to school	0 (0%)	0 (0%)	0 (0%)	0 (0%)	0 (0%)					
Harmony P/C	Yes	9 (81.8%)	58 (82.9%)	1 (50%)	1 (100%)	2 (100%)	6.720	0.567	0.94 (0.18–4.89)	0.01 (1)	0.945
	No	1 (9.1%)	4 (5.7%)	1 (50%)	0 (0%)	0 (0%)					
	Sometimes	1 (9.1%)	8 (11.4%)	0 (0%)	0 (0%)	0 (0%)					
	I do not know	0 (0%)	0 (0%)	0 (0%)	0 (0%)	0 (0%)					
	I do not go to school	0 (0%)	0 (0%)	0 (0%)	0 (0%)	0 (0%)					
Acanth N (Available cases/total cases = 78/86)	Yes	5 (50%)	19 (30.2%)	1 (50%)	1 (100%)	1 (50%)	3.906	0.419	0.48 (0.13–1.83)	1.20 (1)	0.273
	No	5 (50%)	44 (69.8%)	1 (50%)	0 (0%)	1 (50%)					

*p < 0.05; χ² (df) = Pearson Chi-square test statistic with degrees of freedom, tests are Chi-square or Fisher’s exact where appropriate; percentages calculated from available data. *OR* odds ratio, *CI* confidence interval

Table (5) presents a comparison between patients who reported family refusal and those who did not across several psychosocial and clinical variables. Statistically significant associations were observed in bullying experiences (family, peers, and school), with patients experiencing family refusal more frequently reporting bullying across these domains (*p* < 0.05). Similarly, significant differences emerged regarding exposure to sexual exploitation through photos, sadness, and the presence of acanthosis nigricans, all of which were more common in those reporting family refusal. These findings suggest a strong psychosocial and health burden linked to family rejection. However, not all domains demonstrated significant differences. Harmony between parents and children did not vary meaningfully across groups (*p* = 0.674), indicating that family refusal may not necessarily disrupt broader family harmony. While the results highlight clear areas of vulnerability, the relatively small number of patients in the “refusal” and “sometimes” groups warrants cautious interpretation.

*Bullying at school* was reported in 10 of 11 children who refused school (90.9%) compared with 11 of 75 children who attended school (14.7%), showing a statistically significant association (χ² = 30.21, *p* = 0.000; Fisher’s exact *p* = 0.000). The odds of school refusal were approximately 58 times higher among children who experienced bullying at school (OR = 58.18; 95% CI: 6.76–500.92).

*Bullying by peers* was reported in 3 of 11 children who refused school (27.3%) compared with 3 of 75 children who attended school (4.0%), showing a statistically significant association (χ² = 8.01, *p* = 0.005; Fisher’s exact *p* = 0.026). The odds of school refusal were approximately 9 times higher among children who experienced peer bullying (OR = 9.00; 95% CI: 1.55–52.27).

*Bullying by family members* was reported in 4 of 11 children who refused school (36.4%) compared with 4 of 75 children who attended school (5.3%), showing a statistically significant association (χ² = 10.95, *p* = 0.001; Fisher’s exact *p* = 0.008). The odds of school refusal were approximately 10 times higher among children who experienced family bullying (OR = 10.14; 95% CI: 2.07–49.67).

*SE sadness* was reported in 8 of 11 children who refused school (72.7%) compared with 22 of 75 children who attended school (29.3%), showing a statistically significant association (χ² = 7.95, *p* = 0.005; Fisher’s exact *p* = 0.014). The odds of school refusal were approximately 6.4 times higher among children with low self-esteem compared with those with normal self-esteem (OR = 6.42; 95% CI: 1.56–26.50).

*SE Photo* was reported in 5 of 11 children who refused school (45.5%) compared with 7 of 75 children who attended school (9.3%), showing a statistically significant association (χ² = 10.43, *p* = 0.001; Fisher’s exact *p* = 0.007). The odds of school refusal were approximately 8 times higher among children with low photo self-esteem (OR = 8.10; 95% CI: 1.96–33.46).

**Table 5:** Comparison between patients who had refusal of family and who did not as regard bullying patterns, SE photo, sadness, harmony and the presence of acanthosis nigricans (expressed in number and percentage):

		Refusal of family			X^2^	*p* value	OR (95% CI)	χ² (df)	*p* value
		Yes (*n* = 8)	No (*n* = 74)	Sometimes (*n* = 4)					
Bullying in family	Yes	5 (62.5%)	15 (20.3%)	1 (25%)	37.678	0.000*	6.458 (1.394–29.923)	6.931 (1)	0.008*
	No	3 (37.5%)	56 (75.7%)	0 (0%)					
	Sometimes	0 (0%)	3 (4.1%)	3 (75%)					
	I do not know	0 (0%)	0 (0%)	0 (0%)					
	I do not go to school	0 (0%)	0 (0%)	0 (0%)					
Bullying pears	Yes	4 (50%)	2 (2.7%)	0 (0%)	51.981	0.000*	38.000 (5.284–273.254)	25.157 (1)	0.000*
	No	4 (50%)	70 (94.6%)	2 (50%)					
	Sometimes	0 (0%)	1 (1.4%)	2 (50%)					
	I do not know	0 (0%)	1 (1.4%)	0 (0%)					
	I do not go to school	0 (0%)	0 (0%)	0 (0%)					
Bullying in school	Yes	6 (75%)	14 (18.9%)	1 (25%)	12.644	0.049*	12.600 (2.310–68.729)	12.228 (1)	0.000*
	No	2 (25%)	55 (74.3%)	3 (75%)					
	Sometimes	0 (0%)	3 (4.1%)	0 (0%)					
	I do not know	0 (0%)	0 (0%)	0 (0%)					
	I do not go to school	0 (0%)	2 (2.7%)	0 (0%)					
SE photo	Yes	4 (50%)	7 (9.5%)	1 (25%)	10.387	0.034*	8.750 (1.826–41.936)	9.546 (1)	0.002*
	No	4 (50%)	66 (89.2%)	3 (75%)					
	Sometimes	0 (0%)	1 (1.4%)	0 (0%)					
	I do not know	0 (0%)	0 (0%)	0 (0%)					
	I do not go to school	0 (0%)	0 (0%)	0 (0%)					
SE sadness	Yes	6 (75%)	22 (29.7%)	2 (50%)	11.618	0.020*	6.750 (1.269–35.892)	6.249 (1)	0.012*
	No	0 (0%)	44 (59.5%)	1 (25%)					
	Sometimes	2 (25%)	8 (10.8%)	1 (25%)					
	I do not know	0 (0%)	0 (0%)	0 (0%)					
	I do not go to school	0 (0%)	0 (0%)	0 (0%)					
Harmony P/C	Yes	7 (87.5%)	61 (82.4%)	3 (75%)	2.339	0.674	1.531 (0.174–13.460)	0.150 (1)	0.699
	No	1 (12.5%)	5 (6.8%)	0 (0%)					
	Sometimes	0 (0%)	8 (10.8%)	1 (25%)					
	I do not know	0 (0%)	0 (0%)	0 (0%)					
	I do not go to school	0 (0%)	0 (0%)	0 (0%)					
Acanth N (Available cases/total cases = 78/86)	Yes	5 (71.4%)	19 (28.4%)	3 (75%)	8.233	0.016*	0.180 (0.032–0.998)	4.605 (1)	0.032*
	No	2 (28.6%)	48 (71.6%)	1 (25%)					

*p < 0.05; χ² (df) = Pearson Chi-square test statistic with degrees of freedom, tests are Chi-square or Fisher’s exact where appropriate; percentages calculated from available data. *OR* odds ratio, *CI* confidence interval

The results in Table (6) highlight several important associations between refusal of play and psychosocial as well as clinical variables. Statistically significant differences were observed for bullying in the family (*p* = 0.008), bullying by peers (*p* < 0.001), SE photo (*p* = 0.001), and SE sadness (*p* = 0.003). These findings suggest that children who refused play were more likely to experience bullying, both within the family and from peers, and also showed higher tendencies toward sadness and sensitivity regarding photos, reflecting psychosocial distress. Conversely, bullying at school (*p* = 0.890), harmony with parents/children (*p* = 0.639), and acanthosis nigricans (*p* = 0.439) did not differ significantly across groups, indicating that refusal of play is not uniformly associated with all forms of bullying, interpersonal harmony, or dermatological manifestations. While the small number of children who reported refusal of play (*n* = 3) warrants cautious interpretation, the significant associations highlight the need to further investigate bullying and emotional well-being as potential contributors to play refusal.

*Bullying by family members*: Children who experienced bullying within their family were significantly more likely to have family refusal (χ² = 6.93, *p* = 0.008). The odds of family refusal were approximately 6.5 times higher in children experiencing family bullying compared with those who did not (OR = 6.46; 95% CI: 1.39–29.92).

*Bullying at school*: Children who experienced school bullying were significantly more likely to have family refusal (χ² = 12.23, *p* < 0.001). The odds of family refusal were about 12.6 times higher among children who were bullied at school (OR = 12.60; 95% CI: 2.31–68.73).

*Bullying by peers*: Peer bullying was strongly associated with family refusal (χ² = 25.16, *p* < 0.001). Children experiencing peer bullying had 38 times higher odds of family refusal (OR = 38.00; 95% CI: 5.28–273.25).

*Photo SE*: Children with low self-esteem related to appearance were more likely to experience family refusal (χ² = 9.55, *p* = 0.002). Odds of family refusal were 8.75 times higher (OR = 8.75; 95% CI: 1.83–41.94).

*SE sadness*: Low self-esteem was associated with family refusal (χ² = 6.25, *p* = 0.012). Odds were 6.75 times higher in children with low self-esteem (OR = 6.75; 95% CI: 1.27–35.89).

*Acanthosis Nigricans*: Interestingly, children with acanthosis nigricans were less likely to experience family refusal (χ² = 4.61, *p* = 0.032). The odds of family refusal were reduced (OR = 0.18; 95% CI: 0.03–0.998).

**Table 6:** Comparison between patients who had refusal play and who did not as regard bullying patterns, SE photo, sadness, harmony and the presence of acanthosis nigricans (expressed in number and percentage):

		Refusal of play				X^2^	*p* value	OR (95% CI)	χ² (df)	*p* value
		Yes (*n* = 3)	No (*n* = 80)	Sometimes (*n* = 2)	I do not know (*n* = 1)					
Bullying in family	Yes	2 (66.7%)	18 (22.5%)	1 (50%)	0 (0%)	17.319	0.008*	6.737 (0.579–78.418)	3.006 (1)	0.083
	No	1 (33.3%)	57 (71.3%)	1 (50%)	0 (0%)					
	Sometimes	0 (0%)	5 (6.3%)	0 (0%)	1 (100%)					
	I do not know	0 (0%)	0 (0%)	0 (0%)	0 (0%)					
	I do not go to school	0 (0%)	0 (0%)	0 (0%)	0 (0%)					
Bullying pears	Yes	2 (66.7%)	4 (5%)	0 (0%)	0 (0%)	30.271	0.000*	39.500 (2.928–532.962)	17.065 (1)	0.000*
	No	1 (33.3%)	73 (91.3%)	1 (50%)	1 (100%)					
	Sometimes	0 (0%)	2 (2.5%)	1 (50%)	0 (0%)					
	I do not know	0 (0%)	1 (1.3%)	0 (0%)	0 (0%)					
	I do not go to school	0 (0%)	0 (0%)	0 (0%)	0 (0%)					
Bullying in school	Yes	2 (66.7%)	18 (22.5%)	1 (50%)	0 (0%)	4.308	0.890	6.737 (0.579–78.418)	3.006 (1)	0.083
	No	1 (33.3%)	57 (71.3%)	1 (50%)	1 (100%)					
	Sometimes	0 (0%)	3 (3.8%)	0 (0%)	0 (0%)					
	I do not know	0 (0%)	0 (0%)	0 (0%)	0 (0%)					
	I do not go to school	0 (0%)	2 (2.5%)	0 (0%)	0 (0%)					
SE photo	Yes	3 (100%)	8 (10%)	1 (50%)	0 (0%)	21.893	0.001*	—	19.169 (1)	0.000*
	No	0 (0%)	71 (88.8%)	1 (50%)	1 (100%)					
	Sometimes	0 (0%)	1 (1.3%)	0 (0%)	0 (0%)					
	I do not know	0 (0%)	0 (0%)	0 (0%)	0 (0%)					
	I do not go to school	0 (0%)	0 (0%)	0 (0%)	0 (0%)					
SE sadness	Yes	2 (66.7%)	27 (33.8%)	0 (0%)	1 (100%)	19.683	0.003*	3.929 (0.341–45.217)	1.382 (1)	0.240
	No	0 (0%)	45 (56.3%)	0 (0%)	0 (0%)					
	Sometimes	1 (33.3%)	8 (10%)	2 (100%)	0 (0%)					
	I do not know	0 (0%)	0 (0%)	0 (0%)	0 (0%)					
	I do not go to school	0 (0%)	0 (0%)	0 (0%)	0 (0%)					
Harmony P/C	Yes	3 (100%)	66 (82.5%)	1 (50%)	1 (100%)	4.276	0.639	—	0.657 (1)	0.418
	No	0 (0%)	6 (7.5%)	0 (0%)	0 (0%)					
	Sometimes	0 (0%)	8 (10%)	1 (50%)	0 (0%)					
	I do not know	0 (0%)	0 (0%)	0 (0%)	0 (0%)					
	I do not go to school	0 (0%)	0 (0%)	0 (0%)	0 (0%)					
Acanth N (Available cases/total cases = 78/86)	Yes	1 (50%)	24 (32.9%)	1 (50%)	1 (100%)	2.405	0.439	0.520 (0.031–8.655)	0.215 (1)	0.643
	No	1 (50%)	49 (67.1%)	1 (50%)	0 (0%)					

*p < 0.05; χ² (df) = Pearson Chi-square test statistic with degrees of freedom, tests are Chi-square or Fisher’s exact where appropriate; percentages calculated from available data. *OR* odds ratio, *CI* confidence interval

*Bullying by Peers*: Children who were bullied by peers were much more likely to experience family refusal (χ² = 25.16, *p* < 0.001). The odds of family refusal were 38.00 times higher (OR = 38.00; 95% CI: 5.28–273.25).

For *SE photo*, the OR could not be calculated as the data included a cell with zero counts; however, the association was significant based on Fisher’s Exact Test (*p* = 0.002), indicating that children with low SE photo are significantly more likely to refuse play.

ROC analysis was conducted to evaluate the ability of BMI to differentiate children with sadness. Clinical rationale for deriving BMI cut points is based on the established association between higher BMI and psychological distress, as excess weight may contribute to low self-esteem, social stigma, and emotional difficulties.

**Fig. 1:**
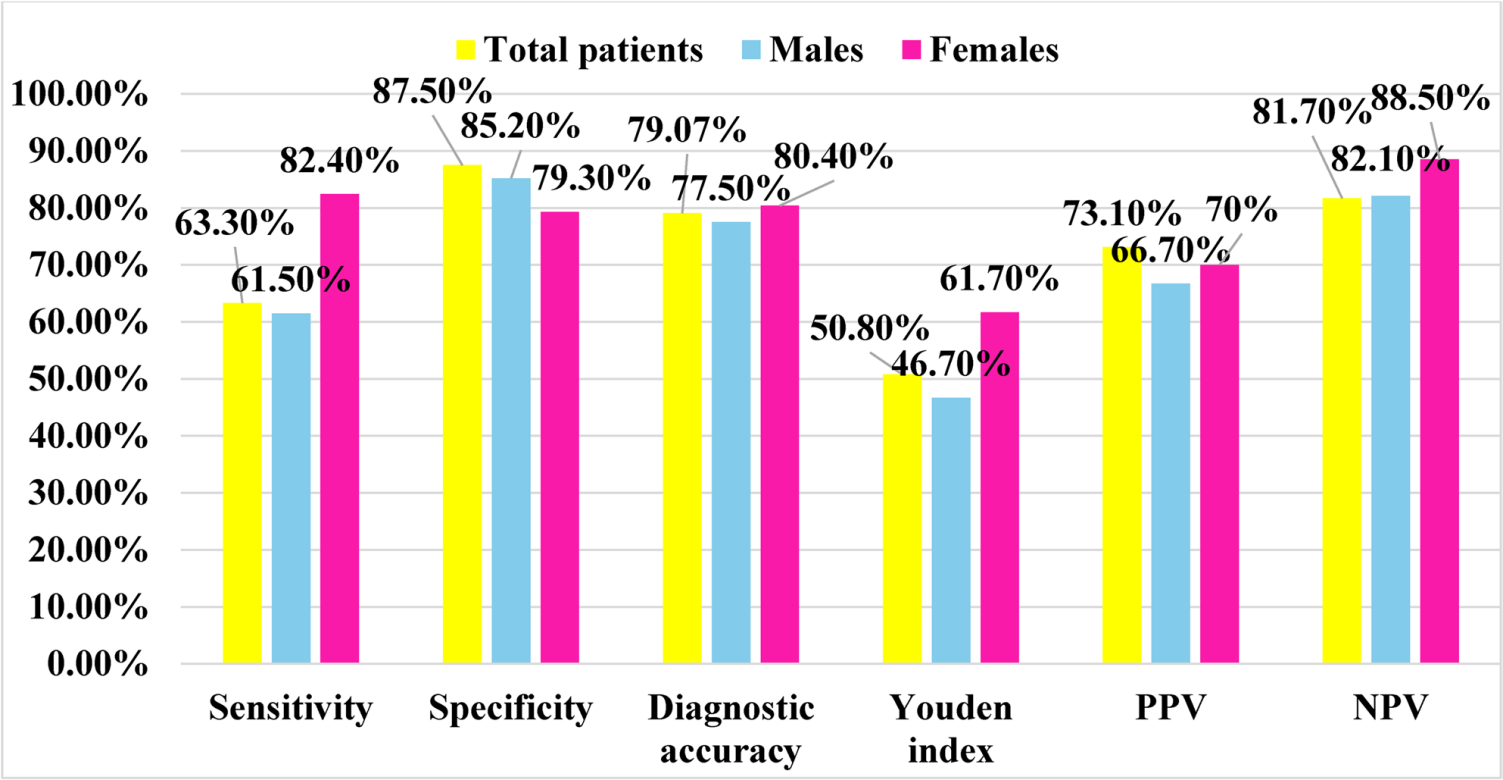
leftacteristics of BMI cut points in differentiating sadness among total patients, males and females

Figures (1) and Table (7) show that the BMI cut point of 25.05 had moderate diagnostic leftacteristics in differentiating the presence of sadness among total patients. The BMI cutoff points for determining the presence of sadness among males and females were 25.1 and 20.85, respectively, indicating more severe affection in female patients than in males.

Youden’s index indicated moderate discriminatory ability for all groups. Calibration of the cut points showed reasonable agreement between predicted and observed proportions of sadness, although caution is warranted because the sample size was small, which may lead to overfitting and limit generalizability.

**Table 7:** Diagnostic performance of BMI in differentiating the presence of sadness among the studied groups:

	AUC	SE	*p*-value	95% CI	Cut-off point
Total patients	0.778	0.057	0.000*	0.666–0.890	25.05
Males	0.714	0.103	0.030*	0.512–0.916	25.1
Females	0.823	0.067	0.000*	0.690–0.955	20.85

*Significant, *AUC* area under curve, *SE *standard error, *CI *confidence interval

Table (8) Multivariable logistic regression analysis was conducted to evaluate independent predictors of sadness. The dependent variable (sadness) was coded as 0 = No and 1 = Yes. Predictors included obesity status (case/control), age (years), and gender. Odds ratios (ORs) with 95% confidence intervals (CI) were estimated. Model fit was assessed using the Omnibus test of model coefficients, Nagelkerke R², classification accuracy, and the Hosmer–Lemeshow goodness-of-fit test.

After adjusting for age and gender, obesity remained a strong independent predictor of sadness among children. Obese participants had approximately 10 folds higher odds of reporting sadness compared with non-obese peers (adjusted OR = 10.14, 95% CI = 3.24–31.78, *p* < 0.001). Neither age (*p* = 0.185) nor gender (*p* = 0.314) significantly predicted sadness. The model was statistically significant (Omnibus χ²(3) = 23.40, *p* < 0.001), explained 33% of the variance (Nagelkerke R² = 0.33), and showed good fit (Hosmer–Lemeshow *p* = 0.486) with 79.1% correct classification.

**Table 8:** Multivariable logistic regression analysis of factors associated with sadness among the studied children (adjusted for age and gender):

Predictor	B	SE	Wald χ² (df = 1)	*p*-value	Adjusted OR (Exp B)	95% CI for OR
Obesity (case/control)	2.317	0.583	15.77	< 0.001*	10.14	3.24–31.78
Age (years)	0.201	0.151	1.76	0.185	1.22	0.91–1.64
Gender (female = 1)	0.538	0.534	1.02	0.314	0.58	0.21–1.66
Constant	−3.384	1.332	6.45	0.011	0.034	—

*p* < 0.05 considered statistically significant. Tests are from the logistic regression model

χ² (df) = Wald Chi-square test statistic with 1 degree of freedom. *OR* odds ratio, *CI* confidence interval

Table (9) After adjusting for age and gender, being in the case group remained a strong independent predictor of family bullying. Case participants had approximately 9 folds higher odds of reporting family bullying compared with controls (adjusted OR = 9.02, 95% CI = 2.34–34.74, *p* = 0.001). Neither age (*p* = 0.771) nor gender (*p* = 0.752) significantly predicted family bullying. The model was statistically significant (Omnibus χ²(3) = 14.66, *p* = 0.002), explained 23% of the variance (Nagelkerke R² = 0.234), and showed good fit (Hosmer–Lemeshow *p* = 0.989) with 75.6% correct classification.

**Table 9:** Multivariable logistic regression analysis of factors associated with bullying in family among the studied children (adjusted for age and gender):

Predictor	B	SE	Wald χ² (df = 1)	*p*-‎value	Adjusted OR (Exp B)	95% CI for OR
Case/control	2.199	0.681	10.42	0.001*	9.02	2.34–34.74
Age (years)	0.045	0.156	0.085	0.771	1.05	0.77–1.42
Gender (female = 1)	0.174	0.552	0.100	0.752	0.84	0.30–2.35
Constant	−2.849	1.386	4.226	0.040	0.058	—

*p* < 0.05 considered statistically significant. Tests are from the logistic regression model

χ² (df) = Wald Chi-square test statistic with 1 degree of freedom. *OR* odds ratio, *CI* confidence interval

Table (10) After adjusting for age and gender, both case/control status and age were significant independent predictors of school bullying. Children in the case group had approximately 4-fold higher odds of experiencing school bullying compared with controls (adjusted OR = 3.94, 95% CI = 1.24–12.53, *p* = 0.020). Older children were also more likely to experience bullying (adjusted OR = 1.47 per year increase, 95% CI = 1.05–2.05, *p* = 0.022). Gender did not significantly predict bullying (*p* = 0.933). The model was statistically significant (Omnibus χ²(3) = 13.18, *p* = 0.004), explained 21% of the variance (Nagelkerke R² = 0.212), showed good fit (Hosmer–Lemeshow *p* = 0.363), and achieved 76.7% correct classification.

**Table 10:** Multivariable logistic regression analysis of factors associated with bullying in school among the studied children (adjusted for age and gender):

Predictor	B	SE	Wald χ² (df = 1)	*p*-value	Adjusted OR (Exp B)	95% CI for OR
Case/control	1.372	0.588	5.44	0.020*	3.94	1.24–12.53
Age (years)	0.385	0.169	5.21	0.022*	1.47	1.05–2.05
Gender (male = 1)	−0.046	0.546	0.007	0.933	0.96	0.34–2.70
Constant	−5.113	1.551	10.86	0.001	0.006	—

*p* < 0.05 considered statistically significant. Tests are from the logistic regression model

χ² (df) = Wald Chi-square test statistic with 1 degree of freedom. *OR* odds ratio, *CI* confidence interval

Table (11) After adjusting for age and gender, case/control status approached significance in predicting school refusal, with case participants having approximately 4.9 times higher odds of school refusal compared with controls (adjusted OR = 4.86, 95% CI = 0.96–24.56, *p* = 0.054). Neither age (*p* = 0.660) nor gender (*p* = 0.719) significantly predicted school refusal. The model was not statistically significant overall (Omnibus χ²(3) = 5.41, *p* = 0.144), explained 11% of the variance (Nagelkerke R² = 0.114), showed acceptable fit (Hosmer–Lemeshow *p* = 0.353), and achieved 87.2% correct classification.

**Table 11:** Multivariable logistic regression analysis of factors associated with refusal of school among the studied children (adjusted for age and gender):

Predictor	B	SE	Wald χ² (df = 1)	*p*-value	Adjusted OR (Exp B)	95% CI for OR
Case/control	1.581	0.821	3.71	0.054	4.86	0.96–24.56
Age (years)	0.084	0.191	0.19	0.660	1.09	0.73–1.62
Gender (male = 1)	0.241	0.671	0.13	0.719	1.27	0.36–4.48
Constant	−3.760	1.742	4.66	0.031	0.023	—

*p* < 0.05 considered statistically significant. Tests are from the logistic regression model

χ² (df) = Wald Chi-square test statistic with 1 degree of freedom. *OR* odds ratio, *CI* confidence interval

## Discussion

Childhood obesity is a growing global public health issue, with its prevalence increasing in both developed and developing nations. Obesity in children is strongly linked to adverse physical and psychological outcomes, including poor body image, increased risk of bullying, and social isolation [17]. This study investigated the relationship between obesity, body image dissatisfaction, and bullying among Egyptian children.

The present study found that obese children had significantly higher BMI values (26.33 ± 4.63) compared to controls (16.65 ± 2.57, *p* < 0.001), along with higher WHTR (0.60 ± 0.06 vs. 0.47 ± 0.05) and higher WHR (median 0.90 [0.87–0.93] vs. 0.87 [0.83–0.90], both *p* = 0.000; Table 1), suggesting central obesity as a defining leftacteristic among the case group. Moreover, 64.0% had WHTR ≥ 0.5 and 31.4% exceeded the 95th percentile in WHR (Table 1), indicating evidence of metabolic health risks. These findings align with global studies that have demonstrated a high prevalence of central obesity among children with BMI above the 95th percentile [18]. A cross-sectional study in China (2020) involving 8130 children aged 7–12 years reported that WHTR ≥ 0.5 was significantly associated with metabolic syndrome, highlighting its utility as a screening tool [19]. Another study in the United States (2018) found that higher BMI and WHTR values were strongly correlated with increased risk of hypertension and type 2 diabetes [20]. The prevalence of acanthosis nigricans (34.6%) in this study further supports these findings, as previous research has shown that this skin condition is a marker of insulin resistance commonly found in obese children [21]. Because blood pressure and acanthosis data had partial missingness (Table 1), prevalence estimates should be interpreted with caution.

Consistent with Tables 2 and 3, obesity was associated with a markedly higher burden of psychosocial adversity. School bullying was significantly more frequent among cases than controls (36.4% vs. 11.9%; χ² = 6.965, *p* = 0.012; OR = 4.24; Table 2), correcting earlier impressions that it only approached significance. Family bullying was also substantially higher among cases (40.9% vs. 7.1%; χ² = 13.275, *p* = 0.000; OR = 9.00; Table 2). These findings are consistent with a meta-analysis of 27 studies published in 2022, which found that obese children were twice as likely to be bullied compared to their normal-weight peers, with family weight-related teasing being a significant contributor to body dissatisfaction [22].

A study conducted by Şahin & Kırlı found that obese children experienced higher rates of verbal and relational bullying than their peers, leading to increased absenteeism and school refusal [23]. In the present study, 20.5% of obese children refused school compared to 4.8% of controls, (*p* = 0.205). However, a significant correlation was found between bullying and school refusal (*p* < 0.000). Although the case–control difference in refusal was not significant (Table 2), bullying correlated strongly with refusal (Table 4): school bullying (OR = 58.18; χ² = 30.21, *p* = 0.000), peer bullying (OR = 9.00; *p* = 0.005), and family bullying (OR = 10.14; *p* = 0.001). Similarly, a systematic review by Cheng et al. found that overweight and obese children were more likely to report school avoidance due to weight-based teasing, with 23% of obese children missing school at least once per week due to bullying [24].

The psychological impact of obesity was evident in this study, sadness much higher among cases than controls (56.8% vs. 11.9%; χ² = 19.083, *p* = 0.000; OR = 9.7; Table 3). Additionally, school refusal correlated with SE-photo and SE-sadness (OR = 8.10 and 6.42, respectively; Table 4). These findings suggest that children who refuse school may develop compensatory mechanisms to protect their self-image or present a distorted body perception. A United Kingdom study with 1,500 children aged 10–14 years found that obese children who experienced weight-based teasing were more likely to develop depressive symptoms and low self-esteem [25]. Similarly, a study by Reiter-Purtill found that self-esteem scores were significantly lower in obese adolescents who reported weight-related victimization, supporting the association between obesity, body dissatisfaction, and mental health [26].

Findings in the present study also showed a gender-based difference in BMI thresholds for sadness, with a BMI cutoff of 25.1 for boys and 20.85 for girls and moderate-to-good discrimination (AUC 0.714 and 0.823, respectively; Table 7; Fig. 1). This aligns with previous research indicating that girls are more susceptible to body dissatisfaction due to societal beauty standards and media portrayals of thinness [27].

Parental and family interactions shape children’s self-esteem and emotional resilience. In this study, children who refused family interactions reported significantly higher rates of bullying across domains (family, peers, school) and higher SE-photo/SE-sadness associations (Table 5), suggesting that a lack of familial support exacerbates victimization in external environments. These findings align with Baggio et al., who explored perspectives on childhood obesity, highlighting that children expressed dissatisfaction with their body image and experienced bullying at school. The study suggests that integrated actions involving health and education professionals, children, and family members are necessary to prevent and combat childhood obesity [28]. A meta-analysis published in 2019 integrated findings from 116 studies to examine the associations between different parenting styles and the self-esteem of children and adolescents. The results indicated that authoritative parenting is positively associated with higher self-esteem. In contrast, authoritarian and neglectful parenting is linked to lower self-esteem in offspring, reinforcing the importance of positive parental reinforcement [29].

Few studies have examined the psychosocial effects of obesity among Egyptian children. A recent study by Hassan et al. reported that a negative correlation was found between bullying victimization and both psychological well-being and self-esteem, suggesting that students who experienced bullying reported more significant emotional distress and lower self-confidence [30]. Similarly, a study conducted in Alexandria found that weight-based teasing in schools was significantly correlated with higher levels of body dissatisfaction, depressive symptoms, and eating disorders [31].

Importantly, the multivariable models (Tables 8, 9, 10 and 11) clarified independence of effects beyond bivariate associations. After adjustment for age and gender, obesity independently predicted sadness (adjusted OR = 10.14; 95% CI: 3.24–31.78; *p* < 0.001; Table 8), accounting for one-third of variance (Nagelkerke R² = 0.33) and showing good fit (Hosmer–Lemeshow *p* = 0.486). Obesity also independently predicted family bullying (adjusted OR = 9.02; 95% CI: 2.34–34.74; *p* = 0.001; Table 9), while both case status and age predicted school bullying (adjusted ORs = 3.94 and 1.47 per year, respectively; Table 10). By contrast, the model for school refusal did not reach statistical significance overall (Omnibus *p* = 0.144), although case status approached significance (adjusted OR = 4.86; *p* = 0.054; Table 11). These patterns reinforce that obesity status is a robust correlate of sadness and bullying—particularly within families and schools—even after accounting for basic demographics.

From a screening perspective, ROC findings (Table 7; Fig. 1) suggest actionable BMI thresholds (≈ 25.05 overall; 25.1 for boys; 20.85 for girls) with moderate discrimination for sadness. While promising for triage, calibration and the modest sample indicate these cut-points should be validated prospectively before clinical adoption.

The findings of this study have significant implications for public health, education, and psychology. Given the strong association between obesity, bullying, and emotional distress, targeted interventions should focus on anti-bullying policies in schools, family-based weight management programs, and psychological support for obese children. Based on Tables 7, 8, 9 and 10, feasible actions include: (i) integrating weight-based teasing into school anti-bullying policies with attention to older age groups at heightened risk; (ii) brief psychosocial screening for sadness among children with obesity (using locally validated tools and piloting BMI-informed triage thresholds); and (iii) family-focused counseling to reduce weight-related teasing at home, given its independent association with bullying and distress. Educational initiatives aimed at reducing weight stigma, promoting healthy body image, and encouraging parental involvement could play a crucial role in mitigating the long-term psychological consequences of childhood obesity [32–34]. Future research should prioritize longitudinal, multi-center designs with fuller measurement of potential confounders (e.g., mental health, sleep, screen time) to test temporality, externally validate the ROC thresholds, and examine whether reducing bullying mediates improvements in mental health among children with obesity.

### Limitations

Reliance on self-reported data may introduce biases, and the study’s focus on family dynamics lacked the psychiatric evaluation of children. Due to the study design, causality cannot be established. Selection bias, related to recruitment from a tertiary outpatient clinic. Social desirability bias, particularly in reporting bullying experiences and emotional states. Expanding outcome measures to include broader health indicators could provide a more comprehensive understanding of obesity related complications and its impact on children and adolescent health.

## Conclusion

This research demonstrates that central obesity is prevalent throughout the study population and serves as a crucial indicator for estimating obesity-related health risks. The presence of metabolic health problems is associated with emotional stress based on this study. School-refusing children demonstrate high levels of emotional distress owing to factors such as school bullying and related educational pressure. School bullying alongside peer victimization produced strong associations to school refusal, while sadness and altered self-perception ratings increased significantly. Negative school social interactions may damage family bonds, whereas broken family relationships might contribute to students experiencing such negative interactions at school. Children who faced family denial experienced higher rates of bullying as well as emotional distress and specific physical health conditions. These findings need further confirmation by additional studies.

## Supplementary Information

Supplementary Material 1.

Supplementary Material 2.

## Data Availability

The datasets used and/or analyzed for the current study are available from the corresponding author on reasonable request.

## Abbreviations

MRCE: Medical research centre of excellence
WHO: World health organization
NRC: National research centre
BMI: Body mass index
CDC: Centre of disease control
ADHD: Attention deficit hyperactivity disorder
WHTR: Waist/Height Ratio
WHR: Waist/Hip Ratio
HTN: Hypertension
SE: Self- esteem
