# Supplementary material for: Body image among bullied obese children: an Egyptian case-control study
Source: BMC Public Health. 2026 Feb 21;26:727. doi: 10.1186/s12889-026-26346-z (PMC12930947; doi:10.1186/s12889-026-26346-z)
Supplement: Supplementary file 1 — Supplementary Material 1. [file 12889_2026_26346_MOESM1_ESM.docx]

| **Questions Directed to the Child's Guardian** | | | | | |
| --- | --- | --- | --- | --- | --- |
|  | Yes | No | Sometimes | I don’t Know |  |
| 1.How is he/she doing at school? Are there any complaints? |  |  |  |  |  |
| a)Especially in reading |  |  |  |  |  |
| b)Especially in mathematics |  |  |  |  |  |
| 2. Has your child refused to go to school due to body image? |  |  |  |  |  |
| 3. Is there bullying at school? |  |  |  |  |  |
| 4. Has he/she refused to attend family gatherings due to body image? |  |  |  |  |  |
| 5. Is there bullying from siblings or relatives? |  |  |  |  |  |
| 6. Has he/she refused to go to the club, participate in activities, or play with other children due to body image? |  |  |  |  |  |
| 7. Is there bullying from neighbors or acquaintances? |  |  |  |  |  |
| 8. Has your child nature changed from being outgoing into introverted? |  |  |  |  |  |
| 9. Does your child refuse to be photographed because of big belly or body shape? |  |  |  |  |  |
| 10. Does he/she talk to you about not being happy with their appearance or clothes? |  |  |  |  |  |
| 11. Does he complains from pain in muscle and bones? |  |  |  |  |  |
| 12.Does he has snoring during sleep? |  |  |  |  |  |
| 13. Does your child wake up several times at night due to breathing difficulty |  |  |  |  |  |
| 14. Are you and your child getting along well? |  |  |  |  |  |

| **Questions Directed to the Child's Guardian (Obesity Case)** | | | |
| --- | --- | --- | --- |
| Since when did you start noticing obesity in your child? | | | |
| Have you noticed signs of early puberty in your child? | | | |
| **Previous Medical Diagnosis For The Child** | | | |
|  | **Yes** | **No** | **I do not know** |
| **Obesity** |  |  |  |
| Has a diet been followed? What is it? |  |  |  |
| Has medication been used to reduce weight? What is it? |  |  |  |
| High blood pressure |  |  |  |
| High fats or cholesterol |  |  |  |
| Fatty liver (previous ultrasound) |  |  |  |
| Kidney problems |  |  |  |
| Diabetes |  |  |  |
| Darkening of skin folds (Acanthosis Nigricans) |  |  |  |
| Obstructive sleep apnea |  |  |  |
| Leg bowing or bone issues? |  |  |  |
| Attention deficit or hyperactivity |  |  |  |
| Learning difficulties |  |  |  |
| Is there any current regular treatment? What is it? |  |  |  |
