# Supplementary material for: Body image among bullied obese children: an Egyptian case-control study
Source: BMC Public Health. 2026 Feb 21;26:727. doi: 10.1186/s12889-026-26346-z (PMC12930947; doi:10.1186/s12889-026-26346-z)
Supplement: Supplementary file 2 — Supplementary Material 2. [file 12889_2026_26346_MOESM2_ESM.docx]

| **Questions Directed to the Child's Guardian** | | | | | |
| --- | --- | --- | --- | --- | --- |
|  | Yes | No | Sometimes | I don’t Know |  |
| 1.How is he/she doing at school? Are there any complaints? |  |  |  |  |  |
| a)Especially in reading |  |  |  |  |  |
| b)Especially in mathematics |  |  |  |  |  |
| 2. Has your child refused to go to school due to body image? |  |  |  |  |  |
| 3. Is there bullying at school? |  |  |  |  |  |
| 4. Has he/she refused to attend family gatherings due to body image? |  |  |  |  |  |
| 5. Is there bullying from siblings or relatives? |  |  |  |  |  |
| 6. Has he/she refused to go to the club, participate in activities, or play with other children due to body image? |  |  |  |  |  |
| 7. Is there bullying from neighbors or acquaintances? |  |  |  |  |  |
| 8. Does your child refuse to be photographed because of his appearance? |  |  |  |  |  |
| 9. Does he/she talk to you about not being happy with their appearance or clothes? |  |  |  |  |  |
| 10. Are you and your child getting along well? |  |  |  |  |  |

| **Previous Medical Diagnosis For The Child** | | |
| --- | --- | --- |
| Is there any medical problems or previous diagnosis for your child? | If yes what is it? | What is treatment given? |
